# The natural selection of words: Finding the features of fitness

**DOI:** 10.1371/journal.pone.0211512

**Published:** 2019-01-28

**Authors:** Peter D. Turney, Saif M. Mohammad

**Affiliations:** 1 Ronin Institute, Montclair, New Jersey, United States of America; 2 National Research Council Canada, Ottawa, Ontario, Canada; University of Edinburgh, UNITED KINGDOM

## Abstract

We introduce a dataset for studying the evolution of words, constructed from WordNet and the Google Books Ngram Corpus. The dataset tracks the evolution of 4,000 synonym sets (*synsets*), containing 9,000 English words, from 1800 AD to 2000 AD. We present a supervised learning algorithm that is able to predict the future leader of a synset: the word in the synset that will have the highest frequency. The algorithm uses features based on a word’s length, the characters in the word, and the historical frequencies of the word. It can predict change of leadership (including the identity of the new leader) fifty years in the future, with an F-score considerably above random guessing. Analysis of the learned models provides insight into the causes of change in the leader of a synset. The algorithm confirms observations linguists have made, such as the trend to replace the -*ise* suffix with -*ize*, the rivalry between the -*ity* and -*ness* suffixes, and the struggle between economy (shorter words are easier to remember and to write) and clarity (longer words are more distinctive and less likely to be confused with one another). The results indicate that integration of the Google Books Ngram Corpus with WordNet has significant potential for improving our understanding of how language evolves.

## Introduction

Words are a basic unit for the expression of meanings, but the mapping between words and meanings is many-to-many. Many words can have one meaning (synonymy) and many meanings can be expressed with one word (polysemy). Generally we have a preference for one word over another when we select a word from a set of synonyms in order to convey a meaning, and generally one sense of a polysemous word is more likely than the other senses. These preferences are not static; they evolve over time. In this paper, we present work on improving our understanding of the evolution of our preferences for one word over another in a set of synonyms.

The main resources we use in this work are the Google Books Ngram Corpus (GBNC) [1–3] and WordNet [4, 5]. GBNC provides us with information about how word frequencies change over time and WordNet allows us to relate words to their meanings.

GBNC is an extensive collection of word ngrams, ranging from unigrams (one word) to five-grams (five consecutive words). The ngrams were extracted from millions of digitized books, written in English, Chinese, French, German, Hebrew, Spanish, Russian, and Italian [1–3]. The books cover the years from the 1500s up to 2008. For each ngram and each year, GBNC provides the frequency of the given ngram in the given year and the number of books containing the given ngram in the given year. The ngrams in GBNC have been automatically tagged with part of speech information. Our experiments use the full English corpus, called *English Version 20120701*.

WordNet is a lexical database for English [4, 5]. Similar lexical databases, following the format of WordNet, have been developed for other languages [6, 7]. Words in WordNet are tagged by their parts of speech and by their senses. A fundamental concept in WordNet is the *synset*, a set of synonymous words (words that share a specified meaning).

**Fig 1:**
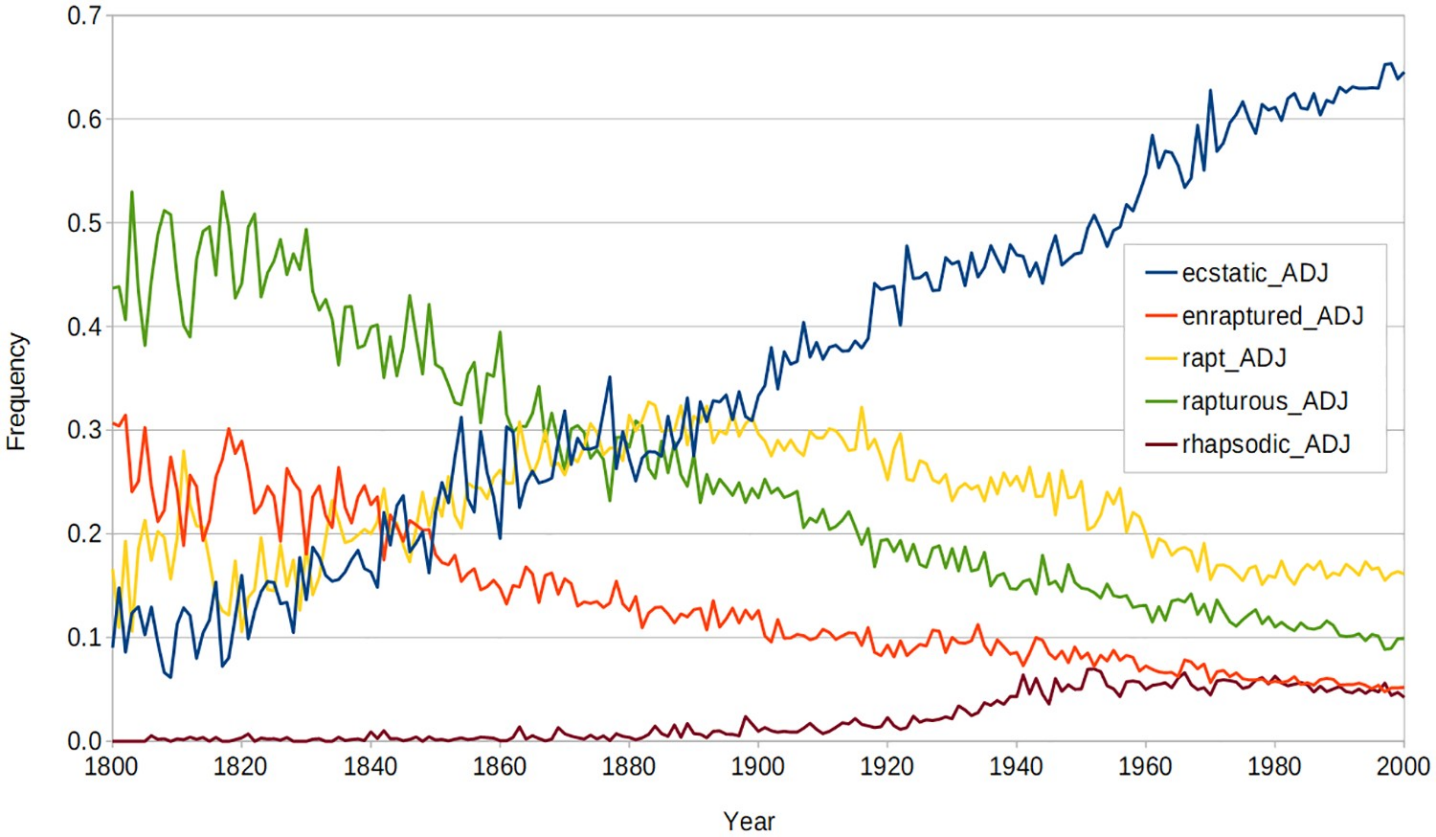
The normalized frequencies of the *rapturous–ecstatic* synset from 1800 AD to 2000 AD. The sum of the five frequencies for any given year is 1.0. The data has not been smoothed, in order to show the level of noise in the trends. This synset is typical with respect to the shapes of the curves and the level of noise in the trends, but it is atypical in that it contains more words than most of the synsets. Most of the synsets contain two to three words.

According to WordNet, *ecstatic*, *enraptured*, *rapt*, *rapturous*, and *rhapsodic* all belong to the same synset, when they are tagged as adjectives (*enraptured* could also be the past tense of the verb *enrapture*). They all mean “feeling great rapture or delight.” Based on frequency information from GBNC, Fig 1 shows that *rapturous* was the most popular member of this synset from 1800 AD to about 1870 AD. After 1870, *ecstatic* and *rapt* competed for first place. By 1900, *ecstatic* was the most popular member of the synset, and its lead over the competition increased up to the year 2000. For convenience, we will refer to this as the *rapturous–ecstatic* synset.

Competition among words is analogous to biological evolution by natural selection. The leading word in a synset (the word with the highest frequency) is like the leading species in a genus (the species with the largest population). The number of tokens of a word in a corpus corresponds to the number of individuals of a species in an environment.

Brandon [8] states that the following three components are crucial to evolution by natural selection:

1. *Variation: There is (significant) variation in morphological, physiological and behavioural traits among members of a species*.
2. *Heredity: Some traits are heritable so that individuals resemble their relations more than they resemble unrelated individuals and, in particular, offspring resemble their parents*.
3. *Differential Fitness: Different variants (or different types of organisms) leave different numbers of offspring in immediate or remote generations*.

Godfrey-Smith [9] lists the same three components, calling them *conditions for evolution by natural selection*.

When a system satisfies these three conditions, we have evolution by natural selection. Synsets satisfy the conditions. There is *variation* in the words in a synset: new words are coined and enter a synset, old words gain new meanings and enter a synset. There is *heredity* in word formation: this heredity is investigated in the field of etymology. There is *differential fitness*: some words become more popular over time and increase in frequency, other words become less popular and decline in frequency. Thus we may say that synsets evolve by natural selection.

Our focus in this paper is on *differential fitness*, also known as *competition* or *selection*. Selection determines which word will dominate (with respect to frequency or population) a synset. Here we do not attempt to model how new words are formed (variation) or how tokens are reproduced with occasional mutations (heredity), although these are interesting topics.

A number of recent papers have examined the problem of understanding how words change their meanings over time [10–13]. In contrast, we examine the problem of understanding how *meanings* (synsets) change their *words* over time. Words compete to represent a meaning, just as living organisms compete to survive in an environment. Regarding competition in biology, Darwin [14] wrote the following:

*[…] it is the most closely allied forms—varieties of the same species and species of the same genus or of related genera—which, from having nearly the same structure, constitution, and habits, generally come into the severest competition with each other*.

Likewise, the words in a synset, having nearly the same meaning, generally come into the severest competition with each other.

The project of understanding how synsets change their leaders raises a number of questions: How much change is due to random events and how much is due to sustained pressures? What are the features of a word that determine its fitness for survival and growth in frequency? Is it possible to predict the outcome of a struggle for dominance of a synset?

We present an algorithm that uses supervised learning to predict the leading member of a synset, applying features based on a word’s length, its letters, and its corpus statistics. The algorithm gives insight into which features cause a synset’s leader to change.

The algorithm is evaluated with a dataset of 4,000 WordNet synsets, containing 9,000 English words and their frequencies in GBNC from 1800 AD to 2000 AD. The dataset enables us to study how English words have evolved over the last two hundred years. In this period, more than 42% of the 4,000 synsets had at least one change in leader. In a typical fifty-year interval, about 16.5% of the synsets experience a change in leader. The algorithm can predict leadership changes (including the identity of the new leader) fifty years ahead with an F-score of 38.5–43.3%, whereas random guessing yields an F-score of 17.3–24.8%.

The main contributions of this paper are (1) the creation and release of a dataset of 4,000 synsets containing a total of 9,000 English words and their historical frequencies [15], (2) a set of features that are useful for predicting change in the leader of a synset, (3) software for processing the dataset with supervised learning [15], generating models that can predict changes in synset leadership, (4) a method for analysis of the learned models that provides insight into the causes of changes in synset leadership.

In the next section, we discuss related work on evolutionary models of word change. The following section describes how we constructed the dataset of synsets and provides some statistics about the dataset. Next, we present the features that we use to characterize the dataset and we outline the learning algorithm. Four sets of experiments are summarized in the subsequent section. We then consider limitations and future work and present our conclusion.

## Related work on the evolution of words

Much has been written about the evolution of words. Van Wyhe [16] provides a good survey of early research. Gray, Greenhill, and Ross [17] and Pagel [18] present thorough reviews of recent work. Mesoudi [19] gives an excellent introduction to work on the evolution of culture in general. In this section, we present a few relevant highlights from the literature on the evolution of words.

Darwin believed that his theory of natural selection should be applied to the evolution of words [20]:

*The formation of different languages and of distinct species, and the proofs that both have been developed through a gradual process, are curiously parallel. […] The survival or preservation of certain favoured words in the struggle for existence is natural selection*.

However, he did not attempt to work out the details of how words evolve.

Bolinger [21] argued that words with similar forms (similar spellings and sounds) should have similar meanings. As an example, he gave the words *queen* and *quean*, the latter meaning “a prostitute or promiscuous woman.” Bolinger claimed the word *quean* has faded away because it violates his dictum.

Magnus [22] defends the idea that some individual phonemes convey semantic qualities, which can be discovered by examining the words that contain these phonemes. For example, several words that begin with the letter *b* share the quality of roundness: *bale, ball, bay, bead, bell, blimp, blip, blob, blotch, bowl, bulb*. We do not pursue this idea here, but we believe resources such as GBNC and WordNet might be used to test this intriguing hypothesis.

Petersen et al. [23] find that, as the vocabulary of a language grows, there is a decrease in the rate at which new words are coined. They observe that a language is like a gas that cools as it expands. Consistent with this hypothesis, we will show that, for English, the rate of change in the leadership of synsets has decreased over time.

Newberry et al. [24] examine three grammatical changes to quantify the strength of natural selection relative to random drift: (1) change in the past tense of 36 verbs, (2) the rise of *do* in negation in Early Modern English, and (3) a sequence of changes in negation in Middle English.

Cuskley et al. [25] study the competition between regular and irregular verbs. They find that the amount of irregularity is roughly constant over time, indicating that the pressures to make verbs conform to rules are balanced by counter-pressures. In our experiments below, we observe rivalries among various suffixes, indicative of similar competing pressures.

Ghanbarnejad et al. [26] analyze the dynamics of language change, to understand the variety of curves that we see when we plot language change over time (as in our Fig 1). They introduce various mathematical models that can be used to gain a deeper understanding of these curves.

Amato et al. [27] consider how linguistic norms evolve over time. Their aim is to distinguish spontaneous change in norms from change that is imposed by centralized institutions. They argue that these different sources of change have distinctive signatures that can be observed in the statistical data.

As we mentioned in the introduction, several papers consider how words change their meanings over time [10–13]. For example, Mihalcea and Nastase [10] discuss the shift in meaning of *gay*, from expressing an emotion to specifying a sexual orientation. Instead of studying how the meaning of a word shifts over time (same word, new meaning), we study how the the most frequent word in a synset shifts over time (same meaning, new word). As Darwin [20] put it, we seek to understand the “preservation of certain favoured words in the struggle for existence.”

## Building datasets of competing words

Predicting the rise and fall of words in a synset could be viewed as a time series prediction problem, but we prefer another point of view. Fifty years from now, will *ecstatic* still dominate its synset, or will it perhaps be replaced by *rapt*? This is a classification problem, rather than a time series prediction problem. The classes are *winner* and *loser*.

Our algorithm has seven steps. The first four steps involve combining information from WordNet and GBNC to make an integrated dataset for studying the competition of words to represent meanings. The last three steps involve supervised learning with feature vectors. We present the first four steps in this section and the last three steps in the section *Learning to Model Word Change*.

The first four steps yield a dataset that is agnostic about the feature vectors and algorithms that might be used to analyze the data. The first step extracts the frequency data we need from GBNC, the second step sums frequency counts for selected time periods, the third step groups words into synsets, and the fourth step splits the data into training and testing sets. The output of the fourth step is suitable for other researchers to use for evaluating their own feature vectors and learning algorithms. The results that other researchers obtain with this feature-agnostic dataset should be suitable for comparison with our results.

In the section *Learning to Model Word Change*, we take as input the feature-agnostic dataset that is the output of the fourth step. The fifth step adds features to the feature-agnostic data, the sixth step applies supervised learning, and the seventh step summarizes the results.

### Past, present, and future

Imagine that the year is 1950 and we wish to predict which member of the *rapturous–ecstatic* synset will be dominant in 2000. In principle, we could use the entire history of the synset up to 1950 to make our prediction; however, it can be challenging to see a trend in such a large quantity of data.

To simplify the problem, we focus on a subset of the data. The idea is, to look fifty years into the future, we should look fifty years into the past, in order to estimate the pace of change. The premise is that this focus will result in a simple, easily interpretable model of the evolution of words.

We divide time into three periods, *past*, *present*, and *future*. Continuing our example, the *past* is 1900, the *present* is 1950, and the *future* is 2000. Suppose that data from these three periods constitutes our testing dataset. We construct the training set by shifting time backwards by fifty years, relative to the testing dataset. In the training dataset, the *past* is 1850, the *present* is 1900, and the *future* is 1950. This lets us train the supervised classification system without peeking into our supposed future (the year 2000, as seen from 1950).

### Integrating GBNC with WordNet

All words in WordNet are labeled with sense information. GBNC includes part of speech information, but it does not have word sense information. To bridge the word sense gap between GBNC and WordNet, we have chosen to restrict our datasets to the monosemous (single-sense) words in WordNet. A WordNet synset is included in our dataset only when every word in the synset is monosemous.

For example, *rapt* is represented in GBNC as *rapt_ADJ*, meaning *the adjective rapt*. We map the GBNC frequency count for *rapt_ADJ* to the WordNet representation *rapt#a#1*, meaning *the first sense of the adjective rapt*. We can do this because the adjective *rapt* has only one possible meaning, according to WordNet. If *rapt_ADJ* had two senses in WordNet, *rapt#a#1* and *rapt#a#2*, then we would not know how to properly divide the frequency count of *rapt_ADJ* in GBNC over the two WordNet senses, *rapt#a#1* and *rapt#a#2*.

The frequency counts in GBNC for the five words *ecstatic_ADJ*, *enraptured_ADJ*, *rapt_ADJ*, *rapturous_ADJ*, and *rhapsodic_ADJ* are mapped to the five word senses *ecstatic#a#1*, *enraptured#a#1*, *rapt#a#1*, *rapturous#a#1*, and *rhapsodic#a#1* in the *rapturous–ecstatic* synset in WordNet. This is permitted because *ecstatic_ADJ*, *enraptured_ADJ*, *rapt_ADJ*, *rapturous_ADJ*, and *rhapsodic_ADJ* are all monosemous in WordNet.

The word *enraptured* could be either an adjective or the past tense of a verb. However, it is not ambiguous when it is tagged with a part of speech, *enraptured_ADJ* or *enraptured_VERB*. Thus we can map the frequency count for *enraptured_ADJ* in GBNC to the monosemous *enraptured#a#1* in WordNet.

There are other possible ways to bridge the word sense gap between GBNC and WordNet. We will discuss this in the section on future work.

### Potential limitations of GBNC and WordNet

Before we explain how we combine GBNC and WordNet, we should discuss some potential issues with these resources. We argue that the design of our experiments mitigates these limitations.

The corpus we use, *English Version 20120701*, contains a relatively large portion of academic and scientific text [28]. The word frequencies in this corpus are not representative of colloquial word usage. However, in our experiments, we only compare relative frequencies of words within a synset. The bias towards scientific text may affect a synset as a whole, but it is not likely to affect relative frequencies within a synset, especially since we restrict our study to monosemous words. The benefit of using *English Version 20120701*, compared to other more colloquial corpora, is its large size, which enables greater coverage of WordNet words and more robust statistical analysis.

Monosemous words tend to have lower frequencies than polysemous words, thus restricting the study to monosemous words creates a bias towards lower frequency words. This could have a quantitative impact on our results. For example, lower frequency words may change more rapidly than higher frequency words, so the rate of change that we see with monosemous words may not be representative of what we would see with polysemous words. Even though this may have an impact on the specific numerical values we report, the same evolutionary mechanisms apply to both the monosemous and polysemous words, and thus the broader conclusions on the trends reported here should be common to both.

In this study, we assume that WordNet synsets are stable over the period of time we consider, 1800 to 2000. Perc [29] argues that English evolved rapidly from 1520 to 1800 and then slowed down from 1800 to 2000. Although it is possible that WordNet synsets may be missing some words that were common around 1800, WordNet appears to have good coverage for the last 200 years. Inspection of WordNet shows that it contains many archaic words that are rarely used today, such as *palfrey* and *paltering*.

Aside from potentially missing words, another possibility is that a word may have moved from one synset to another in the last 200 years. WordNet might indicate that such a word is monosemous and belongs only in the later synset. Although this is possible, we do not know of any cases where this has happened. It is likely that such cases are relatively rare and would have little impact on our conclusions.

### Building datasets

We build our datasets for studying the evolution of words in four steps, as follows.

**Step 1**: *Extract WordNet unigrams from GBNC*. For the first sense of each unigram word in WordNet, if it contains only lower case letters and has at least three letters (for example, *ecstatic#a#1*), then we look for the corresponding word in GBNC (*ecstatic_ADJ*) and find its frequency for each year. GBNC records both the number of tokens of a word and the number of books that contain a word. By *frequency*, we mean the number of tokens. In Step 3, when we group words into synsets, we will eliminate synsets that contain words with a second sense (that is, words that are not monosemous). We believe that, if a synset contains a word that is not monosemous, then it is best to avoid the whole synset, rather than merely removing the polysemous word from the synset.

**Step 2**: *Sum the frequency counts for selected time periods*. GBNC has data extending from 1500 AD to 2008 AD, but the data is sparse before 1800. We sample GBNC for frequency information every fifty years from 1800 to 2000. To smooth the data, we take the sum of the frequency counts over an eleven-year interval. For example, for the year 1800, we take the sum of the frequency counts from 1795 to 1805; that is, 1800 ± 5. Frequency information from 1806 to 1844, from 1856 to 1894, and so on, is not used. (See the first column of Table 1).

**Table 1:** Time periods for the training and testing sets, given a fifty-year cycle of eleven-year samples. The average synset contains 2.23 to 2.25 words.

Period	Train1	Test1	Train2	Test2
1800 ± 5	past			
1850 ± 5	present	past	past	
1900 ± 5	future	present	present	past
1950 ± 5		future	future	present
2000 ± 5				future
Synsets	2,528	3,484	3,484	4,092
Words	5,640	7,795	7,795	9,198
Words per synset	2.23	2.24	2.24	2.25
Change	17.3%	19.0%	19.0%	13.3%

**Step 3**: *Group words into synsets*. Each synset must contain at least two words; otherwise there is no competition between words. Every word in a synset must be monosemous. If any word in a given synset has two or more senses, the entire synset is discarded.

**Step 4**: *Split the data into training and testing sets*. Each training or testing set covers exactly three time periods: *past*, *present*, and *future*. Each training set is shifted fifty years backward from its corresponding testing set. Given a sampling cycle of fifty years, from 1800 to 2000, we have two train–test pairs, as shown in Table 1. We remove a synset from a training or testing set if there is a tie for first place in the *present* or the *future*. We also remove a synset from a training or testing set if it contains any words that are unknown in the *present*. A word is considered to be unknown in the *present* if it has a frequency of zero in the *present*. If a word’s frequency in the *present* is zero, then it is effectively dead and it is not a serious candidate for being a future winner. We decided that including synsets that contain dead words would artificially inflate the algorithm’s score.

The bottom rows of Table 1 give some summary statistics. *Synsets* is the number of synsets in each dataset and *words* is the number of words. *Change* is the percentage of synsets where the leader changed between the *present* and the *future*.

Table 2 shows a sample of the output of Step 4. The sample is the entry for the *rapturous–ecstatic* synset in the Test1 dataset. In 1850 (considered to be the past in Test1), *rapturous* was the leading member of the synset. In 1900 and 1950 (considered to be the present and the future in Test1), *ecstatic* took over the leadership.

**Table 2:** A sample of the Test1 dataset entries for the *rapturous–ecstatic* synset. The highest frequencies for each time period are marked in bold, indicating the winners.

Test1 dataset Ecstatic synset	Past frequency 1850 ± 5	Present frequency 1900 ± 5	Future frequency 1950 ± 5
ecstatic#a#1	5,576	**21,716**	**30,829**
enraptured#a#1	4,334	7,148	5,263
rapt#a#1	5,243	18,750	14,845
rapturous#a#1	**8,645**	15,320	9,544
rhapsodic#a#1	45	696	3,595

### The amount of change in the datasets

A key question about how language evolves is how frequently the meanings have new leaders, and how this rate of leadership change itself changes over time. Table 3 summarizes the amount of change, given a cycle of fifty years from 1800 to 2000, with word frequency counts summed over eleven-year intervals. Here we analyze the data after Step 3 and before Step 4. The table shows that 42% of the synsets had at least one change of leadership over the course of 200 years. Since only five periods are sampled (see the first column in Table 1), at most four changes are possible. The bottom row of Table 3 shows that two synsets experienced this maximum level of churn.

**Table 3:** The frequency of synset leadership changes over 200 years, given a fifty-year cycle of eleven-year samples. Change of leadership is common.

≥ *N* changes	Number of synsets	Percent of synsets
≥ 1 change	1,817	42.14%
≥ 2 changes	518	12.01%
≥ 3 changes	65	1.51%
= 4 changes	2	0.05%

The amount of change that we see depends on the cycle length (fifty years) and the interval for smoothing (eleven years). Shorter cycles and shorter smoothing intervals will show more change, but we should also expect to see more random noise. In the setup we have described in this section, we chose relatively long cycles and smoothing intervals, in an effort to minimize noise. By summing over eleven-year intervals and sampling over fifty-year intervals, we greatly reduce the risk of detecting random synset changes. On the other hand, we increase the risk that we are missing true synset changes. In our experiments, we will explore different cycle lengths.

## Learning to Model Word Change

Now that we have training and testing datasets, we apply supervised learning to predict when the leadership of a synset will change. We do this in three more steps, as follows.

**Step 5**: *Generate feature vectors for each word*. We describe how we generate feature vectors for each word in the next section. Our final aim is to make predictions at the level of synsets. For example, given the *past* and *present* data for the *rapturous–ecstatic* synset in Test1 (see Table 2), we want to predict that *ecstatic* will be the leader of the synset in the *future*. To make such predictions, we first work at the level of individual words, then we later move up to the synset level.

**Step 6**: *Train and test a supervised learning system at the word level*. For each word, we need a model that can estimate its future fitness; that is, the number of tokens the word will have in the future, relative to its competition (the other words in the synset). We treat this as a binary classification task, where the two classes are *winner* and *loser*. However, for Step 7, we need to estimate the probability of being a winner, rather than simply guessing the class. Later we will explain how we obtain probabilities. The probability of winning can be interpreted as the estimated future fitness of a word.

**Step 7**: *Summarize the results at the synset level*. Given probabilities for each of the words in the synset, we guess the winner by simply selecting the word with the highest probability of winning. Thus, for each synset, we have one final output: the member of the synset that we expect to be the winner. The probabilities described in Step 6 are more useful for this step than the binary classes, *winner* and *loser*. Probabilities are unlikely to yield ties, whereas binary classes could easily yield two or more winners or zero winners.

### Feature vectors for words

We represent each word with a vector consisting of eight features and the target class. There are two length-based features, three character-based features, and four corpus-based elements (three features and the class). We will first define the features, then give examples of the vectors.

**Feature 1**: *Normalized length* is the number of characters in the given word, divided by the maximum number of characters for any word in the given synset. The idea is that shorter words might be more fit, since they can be generated with less effort. [*length-based, real-valued*]

**Feature 2**: *Syllable count* is the number of syllables in the given word [30]. The intuition behind this feature is that *normalized length* applies best to written words, whereas *syllable count* applies best to spoken words, so the two features may be complementary. [*length-based, integer-valued*]

**Feature 3**: *Unique ngrams* is the set of letter trigrams in the given word that are not shared with any other words in the given synset. This is not a single feature; it is represented by a high-dimensional sparse binary vector. The motivation for this feature vector is that there may be certain trigrams that enhance the fitness of a word. Before we split the given word into trigrams, we add a vertical bar to the beginning and ending of the word, so that prefix and suffix trigrams are distinct from interior trigrams. For example, *ecstatic* becomes |*ecstatic*|, which yields the trigrams |*ec*, *ecs*, *cst*, *sta*, *tat*, *ati*, *tic*, and *ic*|. However, the trigram *ic*| is not unique to *ecstatic*, since it is shared with *rhapsodic*. Likewise, *rapturous* shares its first four letters with *rapt*, so the unique trigrams for *rapturous* must omit |*ra*, *rap*, and *apt*. Also, overlap with *enraptured* means that *ptu* and *tur* are not unique to *rapturous*. The reason for removing shared trigrams is that they cannot distinguish the winner from a loser; we want to focus on the features that are unique to the winner. [*character-based, sparse binary vector*]

**Feature 4**: *Shared ngrams* is the fraction of letter trigrams in the given word that are shared with other words in the given synset. A large fraction indicates that the given word is quite similar to its competitors, which might be either beneficial or harmful for the word. [*character-based, real-valued*]

**Feature 5**: *Categorial variations* is the number of categorial variations of the given word. One word is considered to be a *categorial variation* of another word when the one word has been derived from the other. Often, but not always, the two words have different parts of speech. For example, *hunger_NOUN*, *hunger_VERB*, and *hungry_ADJ* are categorial variations of each other [31, 32]. The calculation of categorial variations takes the birth date of a word into account; that is, the number of categorial variations of a word does not include variations that were unknown at the specified *present* time. A word with many categorial variations is analogous to a species with many similar species in its genus. This suggests that the ancestor of the species was highly successful [33]. [*character-based, integer-valued*]

**Feature 6**: *Relative growth* is the growth of a word *relative* to its synset. Suppose the word *ecstatic* occurs *n* times in the *present*; that is, *n* is the *raw frequency* of *ecstatic* in the *present*. Suppose the total of the raw frequencies of the six words in the *rapturous–ecstatic* synset is *N*. The *relative frequency* of *ecstatic* is *n*/*N*, the frequency of *ecstatic* relative to its synset. Let *f*_1_, *f*_2_, and *f*_3_ be a word’s relative frequencies in the *past*, *present*, and *future*, respectively. Let Δ be *relative growth*, the change in relative frequency from *past* to *present*, Δ = *f*_2_ − *f*_1_. The *relative growth* of *ecstatic* is its relative frequency in the *present* minus its relative frequency in the *past*. If the synset as a whole is declining, the word in the synset that is declining most slowly will be growing *relative* to its synset. [*corpus-based, real-valued*]

**Feature 7**: *Linear extrapolation* is the expected relative frequency of the given word in the *future*, calculated by linear extrapolation from the relative frequency in the *past* and the *present*. Since the time interval from *past* to *present* is the same as the time interval from *present* to *future* (fifty years), linear extrapolation leads us to expect the same amount of change from the *present* to the *future*; that is, *f*_3_ = *f*_2_ + Δ = 2*f*_2_ − *f*_1_. [*corpus-based, real-valued*]

**Feature 8**: *Present age* is the age of the given word, relative to the *present*. We look in GBNC for the first year in which the given word has a nonzero frequency, and we take this year to be the birth year of the word. We then subtract the birth year from the *present* year, where the *present* year depends on the given dataset. In Step 4, we require all words to have nonzero frequencies in the *present*, so the birth year of a word is necessarily before the *present* year. The idea behind this feature is that older words should be more stable. [*corpus-based, integer-valued*]

**Target class**: The *target class* has the value 1 (*winner*) if the given word has the highest frequency in the given synset in the *future*, 0 (*loser*) otherwise. Ties were removed in Step 4, thus exactly one word in the given synset can have the value 1 for this feature. The *class* is the only element in the vector that uses *future* data, and it is only visible to the learning algorithm during training. The time period that is the *future* in the training data is the *present* in the testing data (see Table 1). [*corpus-based, binary-valued*]

Table 4 shows a sample of the output of Step 5. The sample displays the values of the elements in the vectors for *rapturous* and *ecstatic* in the Test1 dataset. *Unique ngrams* is actually a vector with 3,660 boolean dimensions. Each dimension corresponds to a trigram. The four trigrams that we see for *rapturous* in Table 4 have their values set to 1 in the high-dimensional boolean vector. The remaining 3,656 trigrams have their values set to 0.

**Table 4:** A sample of the Test1 vector elements for two of the five words in the *rapturous–ecstatic* synset.

Feature	rapturous#a#1	ecstatic#a#1
Normalized length	0.900	0.800
Syllable count	3	3
Unique ngrams	uro, rou, ous, us|	|ec, ecs, cst, sta, tat, ati, tic
Shared ngrams	0.556	0.125
Categorial variations	3	2
Relative growth	−0.122	0.107
Linear extrapolation	0.119	0.449
Present age	258	213
Target class	0	1

### Supervised learning of probabilities

We use the naive Bayes classifier [34] in Weka [35, 36] to process the datasets. Naive Bayes estimates the probabilities for the target class by applying Bayes’ theorem with the assumption that the features are independent. We chose the naive Bayes classifier because it is fast, robust, it handles a variety of feature types, and the output model is easily interpretable.

The naive Bayes classifier in Weka has a number of options. We used the default settings, which apply normal (Gaussian) distributions to estimate probabilities. The data is split into two parts, feature vectors for which the *class* is 1 and feature vectors for which the *class* is 0. Each feature is then modeled by its mean and variance for each *class* value, assuming a Gaussian distribution. That is, we have two Gaussians for each feature.

## Experiments with modeling change

This section presents four sets of experiments. The first experiment evaluates the system as described above; we call this system NBCP (Naive Bayes Change Prediction). The second experiment evaluates the impact of removing features from NBCP to discover which features are most useful. The third experiment varies the cycle length from thirty years to sixty years. The final experiment takes a close look at the model that is induced by the naive Bayes classifier, in an effort to understand what it has learned.

### Experiments with NBCP

Table 1 tells us that 19.0% of the synsets in Test1 and 13.3% of the synsets in Test2 undergo a change of leadership. In datasets like this, where there is a large imbalance in the classes (81.0–86.7% in class 0 versus 13.3–19.0% in class 1), accuracy is not the appropriate measure of system performance. We are particularly interested in synsets where there is a change of leadership, but these synsets form a relatively small minority. Therefore, as our performance measures, we use precision, recall, and F-score for leadership change, as explained in Table 5.

**Table 5:** The 2 × 2 contingency table for change in the leadership of a synset.

	Condition positive	Condition nagative
Predicted positive	True positive (*tp*) changed & right	False positive (*fp*) stable & wrong
Predicted negative	False negative (*fn*) changed & wrong	True negative (*tn*) stable & right

The term *changed* in Table 5 means that the *present* leader of the given synset is different from the *future* leader of the synset, whereas *stable* means that the *present* and *future* leader are the same. By *right*, we mean that the given algorithm correctly predicted the *future* leader of the synset, whereas *wrong* means that the given algorithm predicted incorrectly. True positive, *tp*, is the number of synsets that experienced a change in leadership (*changed*) and the given algorithm correctly predicted the new leader (*right*). The other terms, *fp*, *fn*, and *tn*, are defined analogously, by their cells in Table 5.

Now that we have the definitions of *tp*, *fp*, *fn*, and *tn* in Table 5, we can define *precision*, *recall*, and *F-score* [37, 38]:

$$\text{precision}=\frac{tp}{tp+fp}$$ (1)

$$\text{recall}=\frac{tp}{tp+fn}$$ (2)

$$\text{F-score}=2\cdot \frac{\text{precision}\cdot \text{recall}}{\text{precision}+\text{recall}}$$ (3)

The F-score is the harmonic mean of precision and recall. For all three of the above equations, we use the convention that division by zero yields zero. The trivial algorithm that guesses there is never a change in leadership will have a *tp* count of zero, and therefore a precision, recall, and F-score of zero. On the other hand, the accuracy of this trivial algorithm would be 81.0–86.7%, which illustrates why accuracy is not appropriate here.

Table 6 shows the performance of the NBCP system on the two testing sets. With 3,484 synsets, the 95% confidence interval for the scores is ± 1.6%, calculated using the Wilson score interval [39]; thus the F-score for the NBCP system (38.5–43.3%) is significantly better than random guessing (17.3–24.8%), due to the much higher precision of the NBCP system, which compensates for the lower recall of NBCP, compared to random.

**Table 6:** Various statistics for NBCP and random systems. All numbers are percentages, except for *number of synsets*.

Statistic	Test1	Test2
Number of synsets	3,484	4,092
Percent changed	19.0	13.3
Percent stable	81.0	86.7
Precision for random	16.9	10.9
Recall for random	46.1	42.4
F-score for random	24.8	17.3
Precision for NBCP	51.0	47.3
Recall for NBCP	31.0	40.0
F-score for NBCP	38.5	43.3

In Table 6, by *random*, we mean an algorithm that simulates probabilities by randomly selecting a real number from the uniform distribution over the range from zero to one. In Step 6, probabilities are calculated at the level of individual words, not at the level of synsets. Consider the synset {*abuzz*, *buzzing*}. The naive Bayes algorithm treats each of these words independently. When it considers *abuzz*, it does not know that *buzzing* is the only other choice. Therefore, when it assigns a probability to *abuzz* and another probability to *buzzing*, it makes no effort to ensure that the sum of these two probabilities is one. It only ensures that the probability of *abuzz* being a *winner* in the future plus the probability of *abuzz* being a *loser* in the future equals one. Our random system follows the same approach. For *abuzz*, it randomly selects a number from the uniform distribution over the range from zero to one, and this number is taken as the probability that *abuzz* will be the winner. For *buzzing*, it randomly selects another number from the uniform distribution over the range from zero to one, and this is the probability that *buzzing* will be the winner. There is no attempt to ensure that these two simulated probabilities sum to one.

### Feature ablation studies

Table 7 presents the effect of removing a single feature from NBCP. The numbers report the F-score when a feature is removed minus the F-score with all features present. If every feature is contributing to the performance of the system, then we expect to see only negative numbers; removing any feature should reduce performance. Instead, we see positive numbers for *syllable count* and *unique ngrams*, but these positive numbers are not statistically significant.

**Table 7:** The drop in F-score when a feature is removed from the NBCP system. Numbers that are statistically significant with 95% confidence are marked in bold. Negative numbers indicate that a feature is making a useful contribution to the system.

Feature	Test1	Test2
Normalized length	0.00	−0.61
Syllable count	0.12	0.03
Unique ngrams	**−3.49**	0.71
Shared ngrams	0.00	0.00
Categorial variations	−0.07	−0.58
Relative growth	−1.43	−0.76
Linear extrapolation	**−2.54**	**−10.08**
Present age	−0.19	−0.29

The numbers in Table 8 report the F-score for each feature alone minus the F-score for random guessing. We expect only positive numbers, assuming every feature is useful, but there is one signficantly negative number, for *shared ngrams* in Test1.

**Table 8:** The F-score of each feature alone minus the F-score of random guessing. Numbers that are statistically significant with 95% confidence are marked in bold. Positive numbers indicate that a feature is better than random guessing.

Feature	Test1	Test2
Normalized length	**6.36**	**6.85**
Syllable count	0.86	**3.48**
Unique ngrams	**1.66**	**3.71**
Shared ngrams	**−2.72**	−0.08
Categorial variations	−0.02	1.22
Relative growth	**3.95**	**10.10**
Linear extrapolation	**9.62**	**23.49**
Present age	**5.01**	**5.52**

Table 8 shows that *linear extrapolation* is the most powerful feature. Comparing Table 7 with Table 8, we can see that the features mostly do useful work (Table 8), but their contribtion is hidden when the features are combined (Table 7). The comparison tells us that the features are highly correlated with each other.

### Experiments with varying time periods

The NBCP system samples GBNC with a cycle of fifty years, as described above and shown in Table 1. In this section, we experiment with cycles from thirty years up to sixty years. Table 9 reports the F-scores for the different cycle times. The dates given are for the *future* period of each testing dataset, since that is the target period for our predictions.

**Table 9:** The effect that varying cycle lengths has on the F-score of NBCP and random guessing.

Cycle	Test1	Test2	Test3	Test4
30 years	1910 ± 5	1940 ± 5	1970 ± 5	2000 ± 5
F-score for NBCP	34.4	40.6	38.4	38.8
F-score for random	21.0	18.0	15.5	12.6
40 years	1920 ± 5	1960 ± 5	2000 ± 5	
F-score for NBCP	34.7	38.3	42.5	
F-score for random	22.7	19.0	16.1	
50 years	1950 ± 5	2000 ± 5		
F-score for NBCP	38.5	43.3		
F-score for random	24.8	17.3		
60 years	2000 ± 5			
F-score for NBCP	39.5			
F-score for random	21.2			

We have restricted our date range to the years from 1800 AD to 2000 AD, due to the sparsity of GBNC before 1800 AD. We require a minimum of four cycles to build one training set and one testing set (see Train1 and Test1 in Table 1). With a sixty-year cycle, the four time periods that we use are 1820, 1880, 1940, and 2000. Only the final period, 2000, is both a *future* period and a *testing* period. With a seventy-year cycle, the four time periods would be 1790, 1860, 1930, and 2000. Therefore we prefer not to extend the cycle past sixty years.

Regarding periods shorter than thirty years, the amount of change naturally decreases as we shorten the cycle period. With less change, prediction could become more difficult. Table 10 shows how the amount of change varies with the cycle period.

**Table 10:** The effect that varying cycle lengths has on the percentage of synsets that have changed leadership from *present* to *future*.

Cycle	Test1	Test2	Test3	Test4
30 years	1910 ± 5	1940 ± 5	1970 ± 5	2000 ± 5
Percent changed	14.7	13.7	11.0	8.4
Number of synsets	3,041	3,622	3,958	4,275
40 years	1920 ± 5	1960 ± 5	2000 ± 5	
Percent changed	17.5	14.5	11.0	
Number of synsets	3,038	3,732	4,203	
50 years	1950 ± 5	2000 ± 5		
Percent changed	19.0	13.3		
Number of synsets	3,484	4,092		
60 years	2000 ± 5			
Percent changed	15.4			
Number of synsets	3,958			

Comparing Table 9 with Table 10, we see that the F-score of random guessing declines as we approach the year 2000 (see Table 9), following approximately the same pace as the decline of the percent of changed synsets (see Table 10). On the other hand, the F-score of NBCP remains relatively steady; it is robust when the percent of changed synsets varies.

In passing, we note that Table 10 suggests the amount of change is decreasing as we approach the year 2000. This confirms the analysis of Petersen et al. [23], mentioned in our discussion of related work.

### Interpretation of the learned models

In this section, we attempt to understand what the learned models tell us about the evolution of words. For each feature, the naive Bayes classifier generates two Gaussian models, one for class 0 (loser) and one for class 1 (winner). Because naive Bayes assumes features are independent, we can analyze the models for each feature independently.

Here we are attempting to interpret the trained naive Bayes models, to gain insight into the role that the various features play in language change. Since the naive Bayes algorithm assumes the features are independent, the trained models cannot tell us anything about interactions among the features. It is likely that there are interesting interactions among the features. We leave the study of these interactions, possibly with algorithms such as logistic regression, for future work. For now, we focus on the individual impact of each feature.

Table 11 shows the means of the Gaussians (the central peaks of the normal distributions) for the losers and the winners for each feature. This table omits *unique ngrams*, since it is a high-dimensional vector, not a single feature. We will analyze *unique ngrams* separately. The table only shows the models for Test1. Test2 follows the same general pattern.

**Table 11:** Analysis of the naive Bayes models for Test1. The *difference* column is the mean of the Gaussian of the winners (class 1) minus the mean of the Gaussian of the losers (class 0). Differences that are statistically significant are marked in bold. Significance is measured by a two-tailed unpaired *t* test with a 95% confidence level. This table omits *unique ngrams*, which are presented in the next table.

Feature	Means of Gaussians			Mean of the winners is …
	Losers	Winners	Difference	
Normalized length	0.9128	0.9087	−0.0041	lower
Syllable count	3.2494	3.2077	−0.0417	lower
Shared ngrams	0.4797	0.4726	−0.0071	lower
Categorial variations	3.3075	3.4432	0.1357	higher
Relative growth	−0.0012	0.0956	**0.0968**	higher
Linear extrapolation	0.2058	0.8408	**0.6350**	higher
Present age	130.0	180.5	**50.5**	higher

The two length-based features, *normalized length* and *syllable count*, both tend to be lower for winning words. This confirms Bolinger’s [21] view that “economy of effort” plays a large role in the evolution of words; brevity is good. On the other hand, *shared ngrams* also tends to be lower, which implies that we prefer distinctive words. This sets a limit on brevity, since there is a limited supply of short words. Brevity is good, so long as words are not too similar.

We mentioned earlier that a word with many *categorial variations* is analogous to a species with many similar species in its genus, which may be a sign of success. This is supported by the naive Bayes model, since the winner has a higher mean for *categorial variations* than the loser.

The table shows that positive *relative growth* is better than negative *relative growth* and a high *linear extrapolation* is better than low, as expected. It also shows a high *present age* is good. In life, we tend to associate age with mortality, but *present age* is the age of a word *type*, not a *token*; it is analogous to the age of a species, not an individual. A species that has lasted for a long time has demonstrated its ability to survive.

*Unique ngrams* is a vector with 3,660 elements in Test1. To gain some insight into this vector, we sorted the elements in order of decreasing absolute difference between the mean of the Gaussian for class 0 and the mean for class 1. Table 12 gives the top dozen trigrams with the largest gaps between the means. The size of the gap indicates the ability of the trigram to discriminate the classes. The *difference* column is the mean of the Gaussian of the winners (class 1) minus the mean of the Gaussian of the losers (class 0). When the difference is positive, the presence of the trigram in a word suggests that the word might be a winner. When the difference is negative, the presence of the trigram in a word suggests that the word might be a loser.

**Table 12:** Analysis of the *unique ngrams* features in the naive Bayes models for Test1. The table lists the top dozen trigrams with the greatest separation between the means. Differences that are statistically significant are marked in bold; all of the differences are significant. Significance is measured by a two-tailed unpaired *t* test with a 95% confidence level.

Trigrams	Means of Gaussians			Presence of the trigram suggests …
	Losers	Winners	Difference	
ize	0.0055	0.0285	**0.0230**	winner
ise	0.0289	0.0083	**−0.0206**	loser
nes	0.0328	0.0134	**−0.0194**	loser
ty|	0.0112	0.0297	**0.0185**	winner
ss|	0.0379	0.0202	**−0.0177**	loser
ity	0.0100	0.0269	**0.0169**	winner
ze|	0.0022	0.0174	**0.0152**	winner
ess	0.0373	0.0229	**−0.0144**	loser
se|	0.0206	0.0083	**−0.0123**	loser
lis	0.0154	0.0032	**−0.0122**	loser
ic|	0.0228	0.0348	**0.0120**	winner
liz	0.0022	0.0115	**0.0093**	winner

Before splitting a word into trigrams, we added a vertical bar to the beginning and end of the word, to distinguish prefix and suffix trigrams from interior trigrams. Therefore the trigram *ty*| in Table 12 refers to the suffix -*ty*.

In the table, we see that a high value for *ty*| or *ity* indicates a winner, but it is better to have low values for *nes*, *ss*|, and *ess*. Thus the naive Bayes model has confirmed the conflict between the suffixes -*ity* and -*ness* [40]: “Rivalry between the two English nominalising suffixes -*ity* and -*ness* has long been an issue in the literature on English word-formation.” Furthermore, the naive Bayes model suggests that -*ness* is losing the battle to -*ity*.

We also see that *ize*, *ze*|, and *liz* are indicative of a winner, whereas *ise*, *se*| and *lis* suggest a loser. There is a trend to replace the suffix -*ise* with -*ize*. This is known as *Oxford spelling*, although it is commonly believed (incorrectly) that the -*ize* suffix is an American innovation [41].

It is interesting to see that the trigram *ic*| suggests a winner, and *ecstatic* eventually became the leader of the *rapturous–ecstatic* synset (see Fig 1). We looked in the Test1 *unique ngrams* vector for *ous* and found that the presence of *ous* suggests a loser. This may explain why *ecstatic* eventually won out over *rapturous*. However, we then need to explain the poor performance of *rhapsodic* (see Fig 1). Looking again in the Test1 *unique ngrams* vector, the trigram *dic* suggests a loser, whereas the trigram *tic* suggests a winner. Although this is consistent with the success of *ecstatic* and the failure of *rhapsodic*, it is not clear to us why -*tic* should be preferred over -*dic*, given the apparent similarity of these suffixes.

## Future work and limitations

Throughout this work, our guiding principle has been simplicity, based on the assumption that the evolution of words is a complex, noisy process, requiring a simple, robust approach to modeling. Therefore we chose a classification-based analysis, instead of a time series prediction algorithm, and a naive Bayes model, instead of a more complex model. The success of our approach is encouraging, and it suggests there is more signal and structure in the data than we expected. We believe that more sophisticated analyses will reveal interesting phenomena that our simpler approach has missed.

In particular, there is much room for more features in the feature vectors for words. We used three types of features: length-based, character-based, and corpus-based. There are most likely other types that we have overlooked, and other instances within the three types. We did a small experiment with phonetic spelling, using the International Phonetic Alphabet, but we did not find any benefit.

As we said in the introduction, the focus of this paper is *selection*. Future work should consider also *variation* and *heredity*, the other two components of Darwinian evolution. There is some past work on predicting variation of words [42].

This general framework may be applicable to other forms of cultural evolution. For example, the market share of a particular brand within a specific type of product is analogous to the frequency of a word within a synset. The fraction of votes for a political party in a given country is another example.

As we discussed earlier in this paper, to bridge the gap between GBNC and WordNet, we restrict our datasets to the monosemous words in WordNet. Another strategy would be to allow polysemous words, but map all GBNC frequency information for a word to the first sense of the word in WordNet. That is, a synset would be allowed to include words that are not monosemous, but we would assign a frequency count of zero to all senses other than the first sense.

WordNet gives a word’s senses in order of decreasing frequency [4]. In automatic word sense disambiguation, a standard baseline is to simply predict the most frequent sense (the first sense) for every occurrence of a word. This baseline is difficult to beat [43]. This could be used as an argument in support of assigning a frequency count of zero to all senses other than the first sense, as a kind of first-order approximation.

We did a small experiment with this strategy, predicting the most frequent sense for every occurrence of a word. Our dataset expanded from 4,000 synsets containing 9,000 English words to 9,000 synsets containing 22,000 English words. The system performance was numerically different but qualitatively the same as the results we reported above. However, we prefer to take the more conservative approach of only allowing monosemous words, since it does not require us to us to assume that we can ignore the impact of secondary senses on the evolution of a synset. The ideal solution to bridging the gap between GBNC and WordNet would be to automatically sense-tag all of the words in GBNC, but this would involve a major effort, requiring the cooperation of Google.

In the section on related work, we mentioned past research concerned with how words change their meanings over time (same word, new meaning) [10–13]. Let’s call this *meaning-change*. Our focus in this paper has been how meanings change their words over time (same meaning, new word; same synset, new leader). Let’s call this *word-change*. These two types of events, meaning-change and word-change, are interconnected.

Consider the case of a word that has two possible senses, and thus belongs to two different synsets. Suppose that the word’s dominant meaning has shifted over time from the first sense to the second sense, which is a case of meaning-change. If the frequency of the word in the first synset becomes sufficiently low and the frequency of the word in the second synset becomes sufficiently hight, then the word will be less likely to cause a reader or listener to be confused when it is used in the second sense. The meaning-change makes the word less ambiguous and thus it becomes a better candidate for expressing the meaning of the second synset. This meaning-change may therefore cause a word-change. The leader of the second synset might be replaced by the less ambiguous candidate word.

This example illustrates how meaning-change might cause word-change. We can also imagine how word-change can cause meaning-change. We expect that future work will take an integrated approach to these two types of change. If the words in GBNC were automatically sense-tagged, it would greatly facilitate this line of research. Here we have alleviated the issue of meaning-change and its impact on word-change by restricting our dataset to monosemous words. However, we have not completely avoided the issue of meaning-change, since monosemous words can have shifts in connotations. There may also be shifts in meanings that WordNet synsets do not capture. We have avoided precisely defining the relation between meaning-change and word-change, leaving this for future work, since there are multiple reasonable ways to define these terms and their relations.

## Conclusion

This work demonstrates that change in which word dominates a synset is predictable to some degree; change is not entirely random. It is possible to make successful predictions several decades into the future. Furthermore, it is possible to understand some of the causes of change in synset leadership.

Of the various features we examined, the most successful is linear extrapolation. From an evolutionary perspective, this indicates that there is a relatively constant direction in the natural selection of words. The same selective pressures are operating over many decades.

We observed that English appears to be cooling; the rate of change is decreasing over time. This might be due to a stable environment, as suggested by Petersen et al. [23]. It might also be due to the growing number of English speakers, which could increase the inertia of English.

This project is based on a fusion of the Google Books Ngram Corpus with WordNet. We believe that there is great potential for more research with this combination of resources.

This line of research contributes to the sciences of evolutionary theory and computational linguistics, but it may also lead to practical applications in natural language generation and understanding. Evolutionary trends in language are the result of many individuals, making many decisions. A model of the natural selection of words can help us to understand how such decisions are made, which will enable computers to make better decisions about language use.
